# GPT-4 as an X data annotator: Unraveling its performance on a stance classification task

**DOI:** 10.1371/journal.pone.0307741

**Published:** 2024-08-15

**Authors:** Chandreen R. Liyanage, Ravi Gokani, Vijay Mago

**Affiliations:** 1 Department of Computer Science, Lakehead University, Thunder Bay, Ontario, Canada; 2 Department of Social Work, Lakehead University, Thunder Bay, Ontario, Canada; 3 School of Health Policy and Management, York University, Toronto, Ontario, Canada; National Institute of Informatics, JAPAN

## Abstract

Data annotation in NLP is a costly and time-consuming task, traditionally handled by human experts who require extensive training to enhance the task-related background knowledge. Besides, labeling social media texts is particularly challenging due to their brevity, informality, creativity, and varying human perceptions regarding the sociocultural context of the world. With the emergence of GPT models and their proficiency in various NLP tasks, this study aims to establish a performance baseline for GPT-4 as a social media text annotator. To achieve this, we employ our own dataset of tweets, expertly labeled for stance detection with full inter-rater agreement among three annotators. We experiment with three techniques: Zero-shot, Few-shot, and Zero-shot with Chain-of-Thoughts to create prompts for the labeling task. We utilize four training sets constructed with different label sets, including human labels, to fine-tune transformer-based large language models and various combinations of traditional machine learning models with embeddings for stance classification. Finally, all fine-tuned models undergo evaluation using a common testing set with human-generated labels. We use the results from models trained on human labels as the benchmark to assess GPT-4’s potential as an annotator across the three prompting techniques. Based on the experimental findings, GPT-4 achieves comparable results through the Few-shot and Zero-shot Chain-of-Thoughts prompting methods. However, none of these labeling techniques surpass the top three models fine-tuned on human labels. Moreover, we introduce the Zero-shot Chain-of-Thoughts as an effective strategy for aspect-based social media text labeling, which performs better than the standard Zero-shot and yields results similar to the high-performing yet expensive Few-shot approach.

## Introduction

Among the Large Language Models (LLMs), Generative Pre-trained Transformer (GPT) series has emerged as a pioneer, showcasing powerful skills on numerous tasks in Natural Language Processing (NLP), such as content generation, completion, translations, and summarization, and many more [1]. However, the ability of GPT models to comprehend and generate human-like text has not only redefined the landscape of NLP applications but also highlights significant capabilities related to handling many human jobs, such as data analysts [2], data evaluators [3, 4], software developers [5, 6], and teaching assistants [7]. Among these potentialities, GPT has demonstrated itself as an effective tool for data annotation across various domains [8–14]. Its ability to understand context, generate coherent content, and follow specific guidelines has made it a versatile data annotator, in labeling a wide range of content from generic to domain-specific text.

Data annotation is the primary step of many NLP tasks. Nevertheless, the process of labeling by skilled human experts proves to be expensive and time-consuming due to the costs associated with labor, tools, and the time needed for training and manual annotation [10, 13]. Furthermore, maintaining a high standard training process through setting perplexity benchmarks and enough foundation of background knowledge is crucial for high-quality labeling outcomes [15]. Due to these requirements, the consideration of substituting human annotators with Artificial Intelligence (AI) tools has become justifiable.

From another perspective, given the emergence of social media as a significant data source for various NLP studies, addressing the challenges posed by the inherent traits of brevity, informality, creativity, and poor grammar in tweets is essential during annotation [15–17]. Additionally, considering that these texts are embedded within the cultural and social context of human ideas, values, and perceptions of the world, comprehending them necessitates a thorough understanding of context and the ability to empathize by adopting different perspectives [14]. Consequently, the examination and annotation of social media texts, especially those pertaining to social debates, will demand specialized annotation capabilities. This prompts the investigation into the potential of GPT-based models to replace human annotation tasks.

In the literature, several studies have explored the role of GPT as a textual data annotator. A recent investigation assessed the performance of GPT-4 in annotating domain-specific multi-label legal text, a task usually requiring individuals well-versed in legal matters for accurate annotation [11]. Utilizing a dataset comprising 256 records with Krippendorff’s inter-annotator agreement of 0.79, this study demonstrated GPT-4’s capacity to achieve results comparable to human annotators when provided with almost the same copy of instructions. Further, they explained the cost-effectiveness of this approach during batch predictions without a major reduction in performance compared to manual labeling. Nevertheless, slight adjustments to the prompts led to decreased model robustness, significantly impacting outcomes. Moreover, the authors engaged in a failed attempt to improve the performance with the Chain-of-Thoughts (CoT) prompting technique. Another approach has developed to label the political affiliation of tweets collected from the USA politicians [14]. The researcher has used 500 records and executed the GPT-4 model 5 times each with different temperature values; 0.2 and 1.0 to gain both the creativeness and robustness during label prediction. This work achieved better results for accuracy, reliability and bias of GPT-4 compared to human coders for a Zero-shot learning classification task.

The authors of another study have explored three methods to employ GPT-3 for data annotation [10]. The initial approach employed a Few-shot prompt to generate labels for unlabeled data, while the second method designed a prompt to guide the GPT-3 model in self-generating label data. In the third approach, a dictionary was used as an external source of knowledge to assist GPT-3 in creating domain-specific labeled data. They conducted experiments using text-davinci-003 and ChatGPT as GPT-3 models, along with Bert-base as the classifier for evaluation. Findings indicated that the first approach yielded subpar results compared to humans in both accuracy and cost, while the third approach achieved higher performance for GPT-3, surpassing both humans and ChatGPT. Another study has investigated the application of GPT-3.5 and GPT-4 in automated psychological text annotation [8]. This evaluated GPT’s capability to label psychological aspects like sentiment, emotions, and offensiveness across 15 datasets encompassing multiple languages. The results revealed GPT’s remarkable performance compared to dictionary-based analysis and comparable performance to fine-tuned machine learning (ML) models.

Besides the inherited complexities of annotating tweets, some labeling tasks, such as sentiment labeling are relatively straightforward as they focus on identifying sentiments that are often expressed explicitly in the text. Whereas stance classification is a more challenging task for humans as it involves determining the author’s position or perspective toward a particular topic or issue as in favor of, against to, or neutral, which is not always explicitly stated in the text [17–19]. In the existing literature, there are limited studies that have engaged in stance labeling by humans and common target topics of their studies are Atheism, Climate change, Feminism, Elections, and the Legalization of abortion [15, 18, 20].

The earliest dataset of English tweets annotated for stance detection became available to the research community quite recently, in 2016 [15]. This dataset consisted of 4870 tweets, and the annotation process was conducted through crowdsourcing using the CrowdFlower platform. This dataset comprises records where over 60% of the annotators had agreed on the majority label. Many recent studies have utilized this dataset in their stance classification tasks [18–20]. Another study has annotated a corpus of French tweets for detecting stances for a fake news recognition problem [16]. They have implemented a novel annotation approach by presenting the tweets to the annotators as a bundle, comprising a root tweet and all thread tweets as children. They argue the advantage of this approach as annotators gain context from whole threads, improving topic consistency and reducing topic-switching during annotation. While those studies have only provided the text of tweets for the annotators, a different study explored utilizing associated metadata to enrich the labeling process [17]. In the context of political stance detection on Twitter, this study has experimented with a novel labeling approach by providing 6 pieces of additional information related to the authors of tweets other than the tweets’ texts.

The latest development in LLMs involves utilizing prompts to train these models with very little or no prior training data. These techniques are known as Few-shot and Zero-shot learning, and the GPT series of models have proven to excel in these learning scenarios [21]. However, research has demonstrated that GPT models are significantly influenced by their prompts, often producing diverse outcomes [11]. The concept of “Chain-of-Thoughts” was introduced through a Few-shot method that involves presenting a series of intermediate steps to explain a given example answer [22]. They conducted experiments using various versions of prompt-based LLMs, including GPT-3, LaMDA, PaLM, UL2 20B, and Codex. Remarkably, the PaLM 540B model achieved outstanding accuracy on the GSM8K benchmark for math word problems with only eight CoT exemplars and this performance was even better than a fine-tuned GPT-3 model. Subsequently, another study has incorporated this mechanism in Zero-shot prompting [23]. In contrast to the original approach, they omitted to provide examples and instead utilized a two-prompt method, adding the instruction “Let’s think step by step” before each answer in the first prompt. Comparing this Zero-shot approach to the original mechanism, they observed improvements in various reasoning tasks, including arithmetic, symbolic, and logical reasoning. They highlight the advantage of exploring Zero-shot knowledge prior to employing manually crafted Few-shot examples. Furthermore, sophisticated training techniques for LLMs, such as Contextualized Critiques with Constrained Preference Optimization (C3PO), have been introduced to enhance adaptability to specific user prompts while addressing the issue of unintended behavior changes in irrelevant contexts. [24].

While existing studies have demonstrated GPT’s effectiveness in data annotation, minimal attention has been paid to its application in social media stance labeling. Notably, there is a clear gap in the literature for the exploration of prompting techniques with minimal annotation instructions and without metadata involvement. This paper addresses this significant gap by exploring advanced prompting techniques, including Zero-shot, Few-shot, and applying Zero-shot Chain-of-Thoughts specifically to this domain. The introduction of the Zero-shot Chain-of-Thoughts strategy in stance classification significantly enhances the model’s adaptability and performance. Furthermore, this research uniquely utilizes a perfectly reliable ground truth for evaluations, ensuring high accuracy and reliability. It conducts a thorough heuristic assessment using a diverse array of classifiers and comprehensive evaluation measurements, establishing a rigorous performance baseline for GPT-4. By benchmarking GPT-4 against human-labeled data, this study provides valuable insights into its strengths and limitations, highlighting its potential for practical use in real-world social media text annotation. Through these contributions, this research advances NLP methodologies, demonstrating the capabilities of GPT-4 in handling nuanced and context-dependent tasks. Specifically, this study fulfills the following objectives:

1. Create and release a labeled X corpus on stance detection.
2. Benchmark the performance of GPT-4 as a data annotator for labeling social media text on stance detection tasks compared to human experts.
3. Investigate the applicability of integrating the Chain-of-Thoughts concept into the prompt design for labeling the stance of social media texts.
4. Conduct a performance comparison among three distinct prompt-designing strategies in the context of annotating the stance of social media texts.

## Methodology

**Fig 1:**
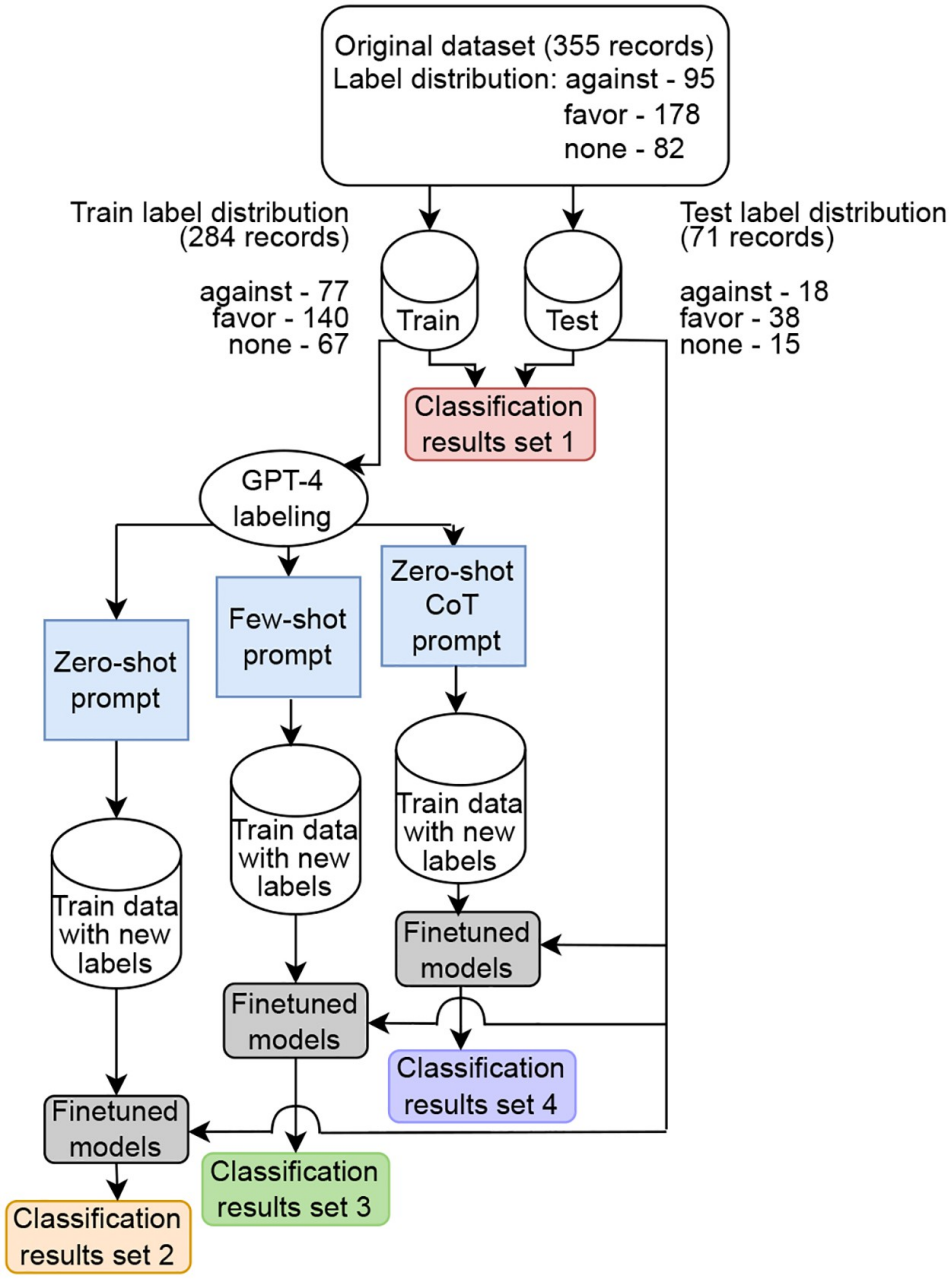
Overall methodology of the study.

Initially, we constructed a labeled corpus of X posts related to the stance classification problem towards abortion legalization. Subsequently, we employed 3 distinct prompting methods to reassign labels to the training tweets using GPT-4. Utilizing these variedly generated labels, along with human annotations, we constructed 4 training datasets containing the same tweets for multi-class classification fine-tuning. Next, the fine-tuned models underwent testing on a shared testing set equipped with human-annotated labels. Finally, we compared the outcomes from the 4 sets of test results to generate comprehensive findings. The complete research methodology is depicted in Fig 1.

### Dataset collection

Motivated by the limited datasets for stance detection, we constructed a dataset by downloading texts related to the topic of abortion legalization from X through X API (Twitter academic API). Focusing on the recent Supreme Court decision to ban abortion in the USA [25], we extracted tweets originating from the USA at three distinct time stamps (TS): i) TS1—before the court decision was leaked (106 days from 16th January 2022 to 1st May 2022), ii) TS2—following the leak (53 days from 2nd May 2022 to 23 June 2022), and iii) TS3—after the court decision (53 days from 24th June 2022 to 15th August 2022), by yielding 250 records from each time stamp. We determined these dates by calculating the number of days between May 2nd (the date of the leak) and June 24th (the date of the court decision). For TS1, we extended the period to twice the duration, as the volume of tweets related to the topic of abortion legalization can be relatively lower. The data collection and analysis methods complied with the X Developer Platform’s terms and conditions by using the official API, observing rate limits, and ensuring the data was used solely for non-commercial research purposes. Additionally, our research adhered to ethical guidelines by solely utilizing publicly available tweets without any interest in or disclosure of author identities, thereby eliminating the need for any ethical considerations related to human subjects, including obtaining participant consent.

### Human data annotation

Under the guidance of a senior academician in Social Science, three postgraduate students underwent specialized training using annotation and perplexity guidelines. Through a series of trial sessions by annotating a few samples, they familiarized themselves with the requirements for achieving a shared understanding. Subsequently, each coder annotated all 750 data points in the corpus for the multi-class stance classification task, regarding the author’s stance on the legalization of abortion as a favor, against, or none. Additionally, the label “uncertain” was provided as an option to indicate instances where annotators are unsure about the suitable label. In our annotation task, we only provided the texts of tweets, omitting their associated metadata. To ensure the reliability of the annotations, we evaluated the results using both Fliess’ Kappa and Krippendorf’s alpha inter-observer agreements [26]. After removing records with at least one uncertain label among annotators, the calculated kappa and alpha were found to be 64.54% and 61.26% respectively. Finally, we employed the majority voting mechanism to finalize the label for each record. We are releasing this dataset of 533 tweets to the public for research purposes [27].

### GPT-4 label generation

As one of the main objectives of our study is to compare GPT-4’s capabilities as an annotator with respect to humans, we needed to utilize reliable baseline labels. As the original dataset shows only substantial agreement among 3 annotators [28], we opted to work with a subset of our corpus, comprising 355 records that achieved 100% inter-reliability agreement among all raters. This subset of data and all the relevant codes are published at [27] to facilitate work replication.

We explored three different prompting strategies: 1) Zero-shot, 2) Few-shot, and 3) Zero-shot with CoT to generate labels for the tweets in our dataset using the specific gpt-4 model (now called gpt-4–0613). We set the temperature as 0.5 which is a lower temperature value as it makes the model more confident in its predictions and leads to more deterministic and focused outputs. However, we did not set the temperature to 0.0, as we needed the model to have some randomness and creativity in predicting our labels [14]. Even though this can help in generating more conservative and precise responses, this will also lead to different answers during different runs. Due to this nature, each prompt type was run 3 times to generate labels for each tweet in the training set and then majority voting was used to finalize the final labels.

#### Zero-shot

**Fig 2:**
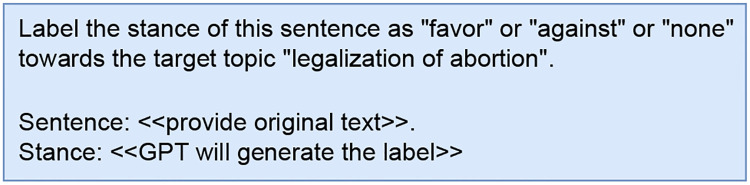
Zero-shot prompt for generating labels.

The first approach is to design a prompt with only instructions (no examples) about the task and provide the tweets without the human-annotated labels in the training set to GPT-4 API call [29]. Within the prompt, we requested the model to produce an appropriate label for the provided text. The prompt design employed for generating labels through the Zero-shot mechanism is illustrated in Fig 2.

#### Few-shot

**Fig 3:**
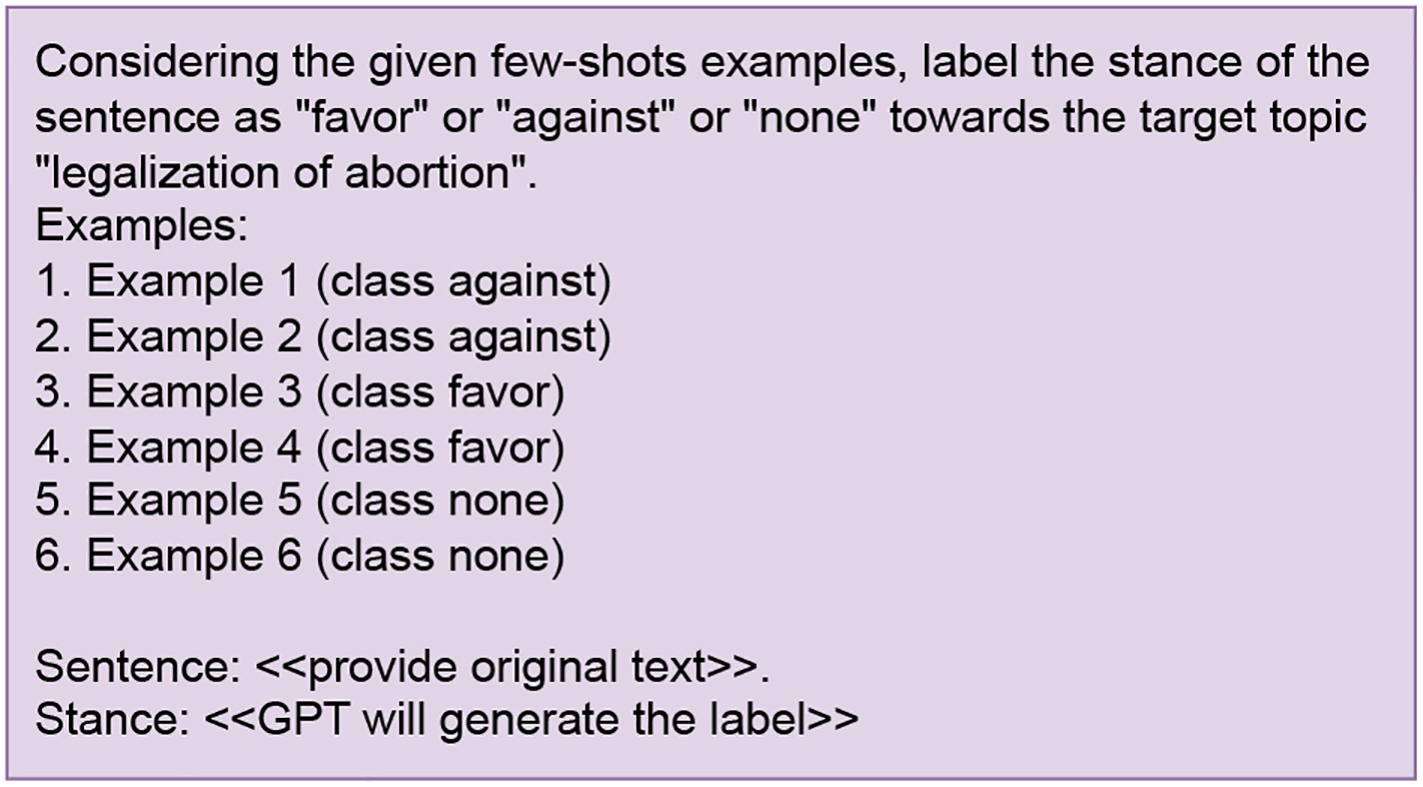
Few-shot prompt for generating labels.

The second method uses a Few-shot learning approach that teaches the GPT-4 model to perform the labeling task utilizing a combination of user instructions and a limited number of examples [29]. To introduce all three classes equally, we provided two fresh examples of tweets and their corresponding human-annotated labels for each class which are mutually exclusive from the training and testing sets (See Fig 3). The Few-shot approach tends to be more expensive compared to the Zero-shot method due to the larger number of tokens in each prompt and the requirement of few samples for the prompt will reduce data from the original dataset.

#### Zero-shot Chain-of-Thought

**Fig 4:**
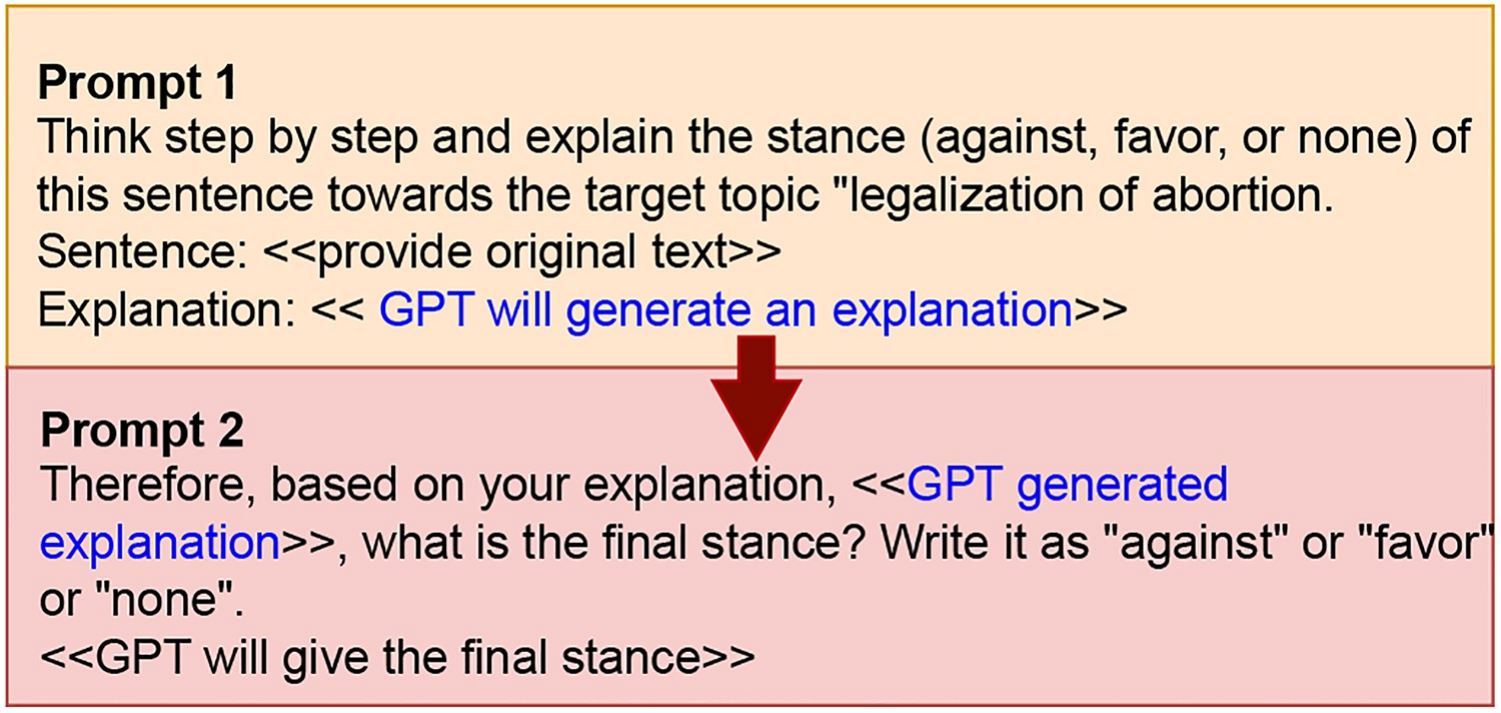
Zero-shot Chain-of-Thoughts prompt for label generation.

This is an extension of Zero-shot prompting where we only provide instructions to the GPT-4 without any examples. The difference between this and the Zero-shot mechanism is that Zero-shot uses only a single prompt and the model will generate the final output at the end. However, as shown in Fig 4, for the concept of Zero-shot CoT, we implemented two prompts, 1) to get a step-by-step explanation of how it decides the author’s stance toward the target topic, and 2) to generate the final stance based on its own explanation. Similar to the original study [23], we instructed the model to think step by step and explain the answer before determining the final stance of the text. Through this two-prompt mechanism, we provide an opportunity for the model to reassess its answer. The advantages of this concept will be further discussed with examples in the “Further discussion” section.

### Stance classification

Stance detection is a multi-class classification problem, often with three stance labels. Our initial dataset with tweets and corresponding human labels was partitioned into an 80:20 ratio as the training and testing sets. Additionally, as mentioned earlier, we generated 3 more training sets featuring the same tweets but with new labels obtained through 3 distinct prompting techniques utilizing GPT-4. Subsequently, we fine-tuned eight transformer-based LLMs, namely Bert [30], Albert [31], Deberta [32], BerTweet [33], MPNet [34], and three Roberta-based models pre-trained on i) a general Twitter dataset (TRob) [35], ii) a Twitter sentiment dataset (TRobSen) [20], and iii) a Twitter stance dataset (TRobStan) [20]. We separately fine-tuned these models using our four training datasets and the list of model versions employed in the study, along with the datasets they were pre-trained on, is provided in S1 Table.

In addition, 18 multiple combinations of classifiers composed of 6 traditional ML models and 3 embedding techniques, namely OpenAI ADA embedding (ADA), Sentence Transformers embedding (SenTr), and Glove embeddings were individually fine-tuned on our 4 training sets. We used embedding techniques to convert the tweets of the training set to their numerical vectors before feeding into the models [36]. Finally, all 104 types of fine-tuned models (32 LLMs and 72 traditional classifiers+embeddings) were tested individually on the common testing set to compare the classification performance of models trained on 4 different label sets.

### Selection of performance metrics

We reported the testing performance in terms of precision, recall, f1-score, Matthews correlation coefficient (MCC) and area under the receiver operating characteristic curve (ROC_AUC). Accuracy was not reported due to its inability to account for class distributions, which makes it unsuitable for evaluating an imbalanced dataset [37, 38].

We used the macro averaging over micro and weighted for calculating precision, recall, f1-score and ROC_AUC as it calculates these metrics for each class independently and then takes the average across all classes. This approach gives equal consideration to all classes, irrespective of their frequency in the dataset. Hence, there is no difference between majority and minority classes, making the evaluations fair for an imbalanced dataset [38]. It is particularly useful in our study as we lack prior knowledge of the real-world class distribution and need to prevent evaluation bias towards dominant classes in different training datasets.

Equations 1–5 in S1 File represent the calculation of precision, recall, and macro averaged precision, recall, and f1-score respectively [38]. The harmonic mean of macro precision and macro recall represents the multi-class macro F1-score.

MCC is a metric ranging between -1 and 1, where a value close to 1 indicates excellent prediction, signifying a robust positive correlation between predicted and actual labels. Conversely, an MCC of 0 signifies no correlation, indicating that the classifier assigns samples to classes randomly, unrelated to their true values. Furthermore, MCC produces negative values, representing an inverse relationship between the predicted and actual classes [37, 38]. For multi-class classification, the MCC can be expressed using equation 6 in S1 File [38].

ROC_AUC is one of the best metrics to measure the performance of imbalanced datasets and it is regarded as a reliable metric, even when dealing with heavily skewed class distributions [39, 40]. For calculating ROC_AUC in multi-class classification, the *TP* rate or *FP* rate is established only after transforming the output into binary form. For this we used the One-vs-Rest (OvR) method to compare each class to all others, treating the others as a single class.

### Hyperparameter tuning

The LLMs underwent fine-tuning using identical hyperparameter configurations: a learning rate of 3e-5, batch size of 16, maximum epochs set at 10 with early stopping based on validation loss, and a patience of 2. Conversely, a grid search was conducted to determine the optimal hyperparameter combinations for traditional ML models. However, for boosting algorithms, we utilized the default setup due to the expected computational complexity associated with hyperparameter evaluation. The traditional models and their corresponding hyperparameter settings are detailed in Table 1. Additionally, a 5-fold cross-validation strategy was employed during model training to mitigate potential overfitting and yield more precise outcomes. Where possible, we employed the “balanced” class weight option to ensure equal significance across all classes to handle class imbalance. All experiments were conducted using a constant random seed value.

**Table 1:** Hyperparameter settings utilized for traditional machine learning models during hyperparameter tuning.

ML model	Hyperparameter settings
Logistic Regression (LR)	‘class_weight’: [None, “balanced”] ‘penalty’: [None, ‘l2’] ‘solver’: [‘lbfgs’, ‘newton-cg’]
Random Forest (RF)	‘n_estimators’: [50, 100, 200] ‘max_depth’: [None, 5, 10] ‘class_weight’: [“balanced”, “balanced_subsample”, None]
Support Vector Classifier (SVC)	‘C’: [1.0, 2.0] ‘class_weight’: [’balanced’, None]
Multi-Layer Perceptron (MLP)	‘activation’: [’logistic’, ‘relu’] ‘solver’: [’sgd’, ‘adam’] ‘hidden_layer_sizes’: [(100,), (200,), (50,)]
Gradient Boosting (GB)	Default settings
Extreme Gradient Boosting (XGB)	Default settings

### Wilcoxon signed-rank test

The Wilcoxon signed-rank test is a fundamental non-parametric statistical test used to compare the central tendencies of paired data or matched samples [41]. This test assesses whether there is a statistically significant difference between two related groups, often before-and-after measurements or two treatments applied to the same subjects. It accomplishes this by analyzing the distribution of the signed differences between the pairs, effectively testing whether the median of these differences is zero [42, 43]. For our study, we used the Wilcoxon signed-rank test [44] to assess and summarize the similarity between performance metrics of various combinations of prompting outcomes.

We utilized the conventional value of 0.05 as the threshold for accepting or rejecting the null hypothesis, which assumes there is no significant difference between the corresponding performance metrics (either, precision, recall, f1-score, or ROC_AUC) of any two labeling sets. Here, in addition to the null hypothesis, we used an alternative hypothesis called ‘greater’ which suggests that the median of the paired differences is greater than zero. This test produces two main outputs, 1) test-statistics—the sum of ranks of positive differences, which measures the extent to which the positive differences between paired observations are greater than the negative differences, and 2) P-value—which determines whether this difference holds statistical significance. Consequently, higher test-statistics (larger positive difference between the two groups) indicate that the first group tends to have higher values than the second group, and the P-values below the selected significance level of 0.05 present there are statistically significant evidence to prove this difference. Eqs (1) and (2) represents the calculation of the test-statistic and P-value of the Wilcoxon signed-rank test with the ‘greater’ alternative hypothesis [45].

- The test-statistic (*W*+):
  $$\begin{array}{c} W+=\sum_{i=1}^{n}sign\left(d_{i}\right).R_{i}^{+}, \end{array}$$ (1)
  where, *n* is the sample size, *di* represents the paired differences, *sign*(*di*) is the sign of the difference (+1 if *di* is positive, -1 if *di* is negative), and *Ri*+ is the rank of the positive differences among all the positive differences.
- The P-value (*P*_*val*):
  $$\begin{array}{c} P-val=P(W+\geq W_{observed}) \end{array}$$ (2)
  Where, *W*+ is the test-statistic calculated from our data, *W*_*observed*_ is the test-statistic from the Wilcoxon signed-rank table [46] (based on the chosen significance level of 0.05 and sample size of 26), and *P* is the probability of observing a *W*+ value greater than or equal to *W*_*observed*_ under the null hypothesis.

## Experimental results and initial discussion

First, we analyze the outcomes of the relabeling process by examining the distribution of class labels in both the original and new label sets. Following this, we present the classification results of various ML models which were fine-tuned using the four distinct training sets.

### Results of label generation

**Fig 5:**
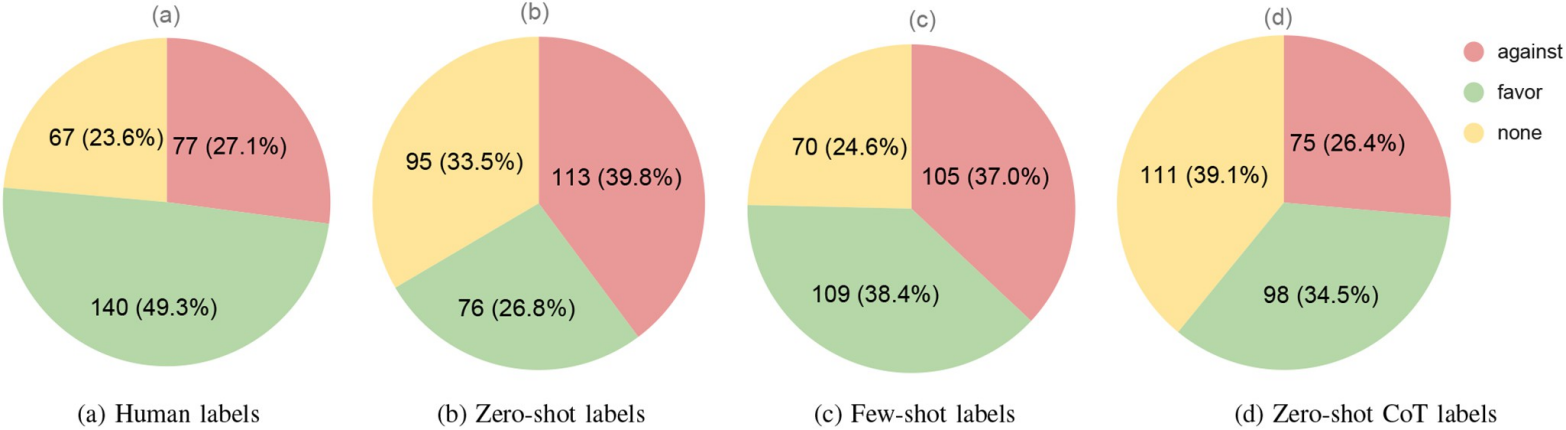
The distribution of class labels in the four different label sets. (a) human labels, (b) Zero-shot labels, (c) Few-shot labels, and (d) Zero-shot CoT labels.

Fig 5 illustrates the distribution of class labels within the four training sets, created using different labeling techniques. Notably, datasets labeled by humans and the Few-shot approach exhibit a similarity, showcasing almost equal ratios in their ‘none’ class and gaining the ‘favor’ as the majority class. However, a significant change has occurred due to the ‘against’ class incrementing to 37% in the Few-shot labeled dataset, resulting in an almost 1:1 ratio with the ‘favor’ class. This contrast stands against the nearly 2:1 ‘favor: against’ ratio seen in the human-labeled dataset. On the other hand, compared to human labels, the Zero-shot and Zero-shot CoT datasets have undergone a shift, with their majority classes changing to ‘against’ and ‘none’, respectively. Furthermore, the ‘favor’ and ‘against’ classes in the Zero-shot and Zero-shot CoT datasets have become the minority respectively, departing from the ‘none’ which served as the minority class in the human-labeled datasets. Nevertheless, the sizes of the ‘against’ class in both the Zero-shot CoT and human-labeled datasets are nearly similar.

**Fig 6:**
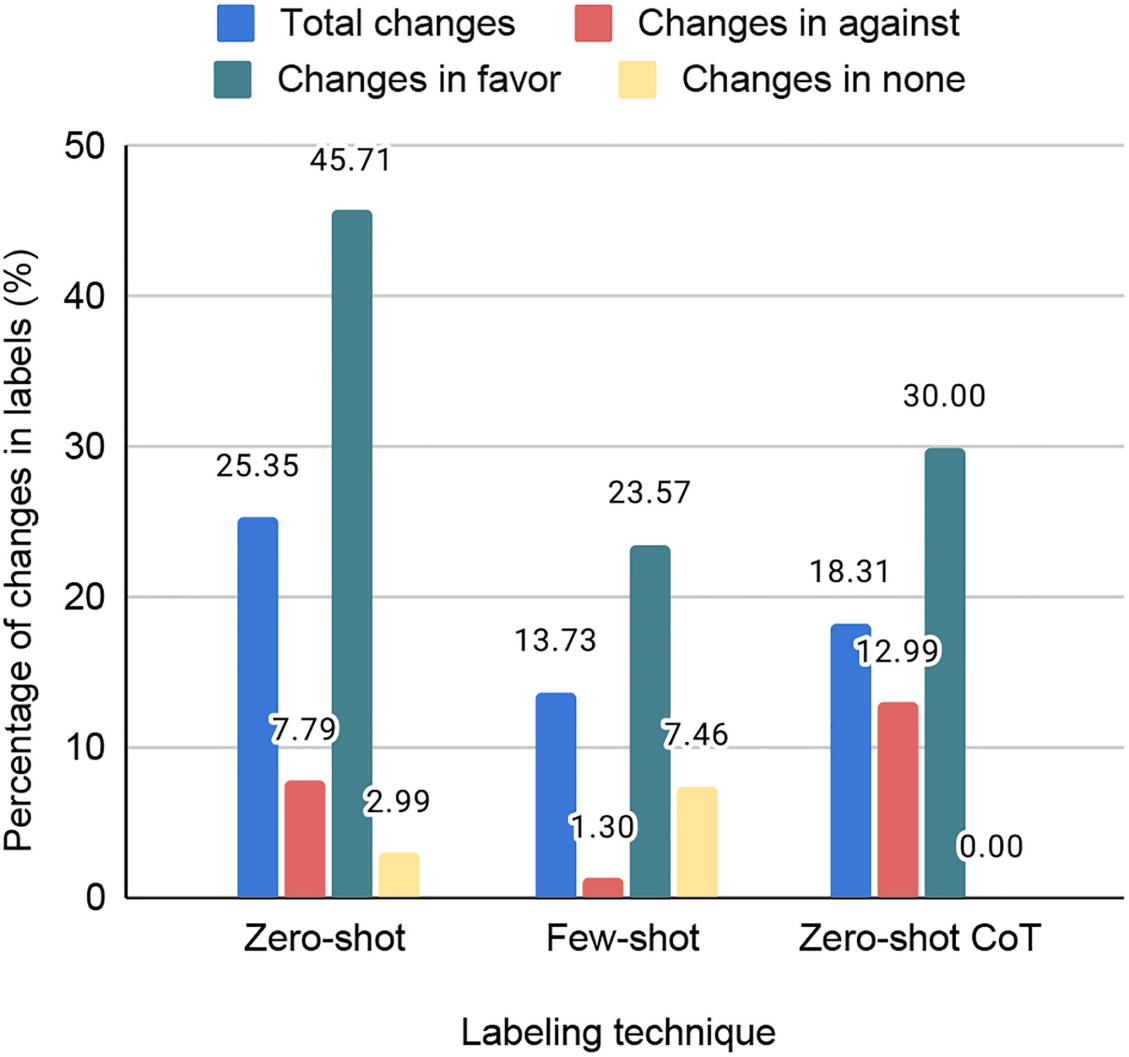
The percentages of changes in the three types of new label sets; Zero-shot, Few-shot, and Zero-shot CoT compared to human labels.

Fig 6 displays the percentage of changes observed with new label sets compared to the human labels. This demonstrates that the highest number of changes in the whole dataset appeared as 25.35% during the Zero-shot approach, whereas a minimum of 13.73% is recorded at the Few-shot. Analyzing class-wise percentages, the ‘favor’ class experienced the highest variations, reaching 45.71%, 23.57%, and 30.0% in the Zero-shot, Few-shot, and Zero-shot CoT methods, respectively. Moreover, the minimum change percentage of the ‘against’ class is recorded as 1.30% in the Few-shot technique, whereas a minimum of 0.0% in the ‘none’ class is reported in the Zero-shot CoT approach.

By considering both the label distribution and the percentage of changes, we observe that, in comparison to the labels generated by the Zero-shot method, both Few-shot and Zero-shot CoT approaches produce labels that are more similar to those generated by humans.

### Classification results

The classification results obtained for five evaluation metrics are shown in Table 2. The rows represent all combinations of classification models, including transformer-based LLMs and combinations of embeddings and traditional ML models. Whereas the main columns represent the four training sets with different labels used to fine-tune these models. By setting the results of models fine-tuned on human labels as the ground truth, we highlighted (in green) the instances of the other three labeling sets that surpassed the corresponding baseline value. Overall, the Few-shot and Zero-shot CoT have obtained better results for many models. According to LLMs’ results, BerTweet; a model pre-trained on 850M English Tweets (See Appendix) has outperformed the ground truth when fine-tuned on Few-shot and Zero-shot CoT labels. Similarly, this model has gained better or equal precision, recall, and MCC when fine-tuned on Zero-shot labels. Besides, MPNet and TRobStan on Few-shot labels, and Bert on Zero-shot CoT labels, have shown remarkable results on various metrics.

**Table 2:** Testing results of models fine-tuned on four training sets with different labels.

Model	Classification results set 1 : Human labels					Classification results set 2 : Zero-shot labels					Classification results set 3 : Few-shot labels					Classification results set 4 : Zero-shot CoT labels				
	pre	rec	f1	mcc	roc	pre	rec	f1	mcc	roc	pre	rec	f1	mcc	roc	pre	rec	f1	mcc	roc
Bert	0.70	0.69	0.69	0.53	0.84	0.64	0.67	0.59	0.44	0.81	0.68	0.61	0.60	0.40	0.83	0.68	0.74	0.69	0.56	0.84
Albert	0.57	0.59	0.54	0.33	0.70	0.44	0.48	0.42	0.15	0.66	0.46	0.41	0.41	0.10	0.65	0.48	0.53	0.46	0.21	0.73
Debert	0.73	0.64	0.67	0.52	0.82	0.63	0.67	0.57	0.44	0.76	0.64	0.63	0.61	0.41	0.81	0.60	0.64	0.59	0.42	0.80
BerTweet	0.72	0.70	0.71	0.55	0.89	0.72	0.76	0.69	0.57	0.86	0.75	0.76	0.72	0.59	0.91	0.76	0.74	0.75	0.62	0.88
MPNet	0.81	0.76	0.77	0.67	0.91	0.69	0.71	0.65	0.49	0.81	0.82	0.79	0.79	0.66	0.95	0.75	0.73	0.74	0.63	0.85
TRob	0.83	0.77	0.79	0.67	0.94	0.78	0.76	0.72	0.58	0.88	0.76	0.75	0.74	0.59	0.92	0.70	0.73	0.71	0.59	0.92
TRobSen	0.82	0.73	0.75	0.62	0.92	0.69	0.68	0.62	0.48	0.85	0.75	0.66	0.64	0.48	0.88	0.72	0.77	0.73	0.61	0.89
TRobStan	0.82	0.74	0.77	0.64	0.89	0.73	0.71	0.69	0.51	0.87	0.82	0.79	0.77	0.66	0.92	0.72	0.75	0.72	0.59	0.90
LR-ADA	0.78	0.77	0.77	0.65	0.92	0.65	0.70	0.63	0.49	0.87	0.76	0.80	0.77	0.66	0.91	0.70	0.75	0.70	0.58	0.90
RF-ADA	0.86	0.65	0.70	0.58	0.86	0.63	0.67	0.58	0.44	0.86	0.73	0.73	0.71	0.56	0.89	0.73	0.70	0.66	0.53	0.88
SVM-ADA	0.81	0.83	0.81	0.71	0.93	0.69	0.73	0.68	0.54	0.88	0.75	0.77	0.74	0.62	0.92	0.71	0.76	0.72	0.60	0.90
MLP-ADA	0.89	0.87	0.88	0.81	0.94	0.62	0.66	0.58	0.43	0.87	0.78	0.79	0.77	0.64	0.92	0.72	0.75	0.71	0.59	0.89
GB-ADA	0.77	0.64	0.67	0.55	0.91	0.68	0.71	0.60	0.50	0.82	0.66	0.65	0.64	0.47	0.86	0.64	0.66	0.61	0.46	0.86
XGB-ADA	0.79	0.71	0.74	0.62	0.91	0.64	0.64	0.54	0.42	0.87	0.68	0.72	0.68	0.52	0.87	0.68	0.69	0.61	0.48	0.88
LR-SenTr	0.74	0.78	0.75	0.63	0.90	0.68	0.68	0.56	0.47	0.85	0.71	0.76	0.71	0.58	0.88	0.71	0.77	0.72	0.60	0.88
RF-SenTr	0.71	0.64	0.66	0.49	0.85	0.64	0.62	0.49	0.39	0.80	0.66	0.67	0.64	0.48	0.86	0.71	0.69	0.66	0.52	0.85
SVM-SenTr	0.71	0.69	0.69	0.53	0.89	0.64	0.66	0.60	0.43	0.84	0.70	0.70	0.67	0.53	0.87	0.70	0.75	0.71	0.58	0.86
MLP-SenTr	0.75	0.69	0.71	0.57	0.91	0.68	0.70	0.63	0.49	0.87	0.77	0.80	0.77	0.65	0.88	0.69	0.72	0.68	0.55	0.87
GB-SenTr	0.63	0.58	0.59	0.39	0.84	0.62	0.63	0.56	0.40	0.80	0.69	0.62	0.60	0.43	0.81	0.68	0.70	0.67	0.54	0.86
XGB-SenTr	0.72	0.68	0.69	0.52	0.88	0.63	0.65	0.56	0.42	0.79	0.69	0.69	0.65	0.49	0.82	0.61	0.65	0.60	0.43	0.81
LR-Glove	0.63	0.60	0.61	0.40	0.80	0.52	0.55	0.52	0.29	0.75	0.59	0.56	0.54	0.32	0.78	0.57	0.62	0.56	0.37	0.77
RF-Glove	0.62	0.54	0.56	0.36	0.80	0.61	0.63	0.54	0.40	0.78	0.59	0.61	0.58	0.43	0.79	0.53	0.56	0.52	0.32	0.75
SVM-Glove	0.58	0.51	0.53	0.28	0.78	0.54	0.53	0.51	0.29	0.74	0.60	0.58	0.56	0.37	0.80	0.53	0.56	0.52	0.30	0.73
MLP-Glove	0.63	0.56	0.58	0.34	0.78	0.51	0.52	0.46	0.25	0.75	0.59	0.56	0.55	0.31	0.78	0.59	0.62	0.58	0.40	0.78
GB-Glove	0.52	0.50	0.51	0.25	0.76	0.48	0.51	0.43	0.24	0.70	0.52	0.52	0.51	0.29	0.71	0.55	0.55	0.53	0.32	0.78
XGB-Glove	0.59	0.55	0.56	0.36	0.77	0.49	0.52	0.45	0.24	0.73	0.58	0.53	0.54	0.29	0.77	0.59	0.62	0.58	0.40	0.79
AVERAGE	0.72	0.67	0.68	0.52	0.86	0.63	0.64	0.57	0.42	0.81	0.68	0.67	0.65	0.48	0.84	0.66	0.68	0.64	0.49	0.84

Noticeably, the traditional ML models have gained surpassing results, when the embedding techniques are Sentence Transformers or Glove. Besides, many of the embedding and traditional ML model combinations, such as Random Forest and Gradient Boosting Tree with Sentence Transformers and Gradient Boosting Tree and XGB classifier with Golve have exceeded the baseline margins when they are trained on Zero-shot CoT labels. However, for Few-shot learning, only SVM with GLove embedding has fully overpassed the human-label performance. On average, we noticed that the recalls of all the models when trained on Few-shot and Zero-shot CoT labels have reached or improved upon the baseline performance.

### Results of Wilcoxon signed-rank test

Next, to summarize and compare the classification results mentioned above, we conducted a Wilcoxon signed-rank test by analyzing the performance metrics of different pairs of labeling sets. The results for each of the six possible pairs of labeling sets are presented in Table 3, showing the corresponding test-statistic and P-values. Here, we calculated the difference between the two groups as (Training label set ‘a’—Training label set ‘b’). The test-statistic values, which are larger and fall within the range of 250 to 350, along with significantly smaller P-values ranging from E-08 to E-02 for precision, f1-score, MCC, and ROC_AUC, indicate that the classification results for H-Z, H-F, and H-ZC are notably better when the models are trained using human labels compared to the corresponding three label types. On the contrary, relatively larger P-values (6.91E-01, 9.25E-01) and smaller test-statistic values (144.0, 119.5) for recall in the H-F and H-ZC comparisons illustrate that the classification results of Few-shot and Zero-shot CoT label types are closer to that of human labels.

**Table 3:** Results of Wilcoxon signed-rank test performed to compare the evaluation metrics of each of two sets of labels generated by different approaches. The ‘W’ refers to the test-statistic and p-val refers to the P-value.

Training label set ‘a’	Training label set ‘b’	Precision		Recall		F1-score		MCC		ROC_AUC	
		W	p-val	W	p-val	W	p-val	W	p-val	W	p-val
Human (H)	Zero-shot (Z)	351.0	1.49E-08	250.0	2.97E-02	351.0	1.49E-08	340.0	8.20E-07	350.0	2.98E-08
Human (H)	Few-shot (F)	282.0	6.50E-04	144.0	6.91E-01	281.5	3.07E-03	262.0	1.36E-02	275.0	5.09E-03
Human (H)	Zero-shot CoT (ZC)	306.0	5.64E-05	119.5	9.25E-01	295.0	8.02E-04	247.0	3.55E-02	276.0	1.12E-03
Zero-shot (Z)	Few-shot (F)	11.5	1.00E+00	78.0	9.89E-01	2.0	1.00E+00	33.5	1.00E+00	6.0	1.00E+00
Zero-shot (Z)	Zero-shot CoT (ZC)	66.5	9.98E-01	43.0	9.99E-01	6.5	1.00E+00	22.0	1.00E+00	19.5	1.00E+00
Few-shot (F)	Zero-shot CoT (ZC)	275.5	5.09E-03	153.0	7.17E-01	213.5	1.77E-01	155.0	7.00E-01	187.0	1.45E-01

When comparing Zero-shot to both Few-shot and Zero-shot CoT performances, it is evident that the test-statistic values are consistently smaller, falling within the range of 2.0 to 78.0. This observation suggests that Zero-shot generally results in smaller values compared to the other two. Furthermore, the larger P-values, which range from E-01 to E+00, indicate that there is no statistically significant evidence to support the claim that Zero-shot tends to yield larger values. This indicates that these two techniques outperform the basic Zero-shot method significantly across all metrics. Based on the larger P-values obtained for the comparison of Few-shot and Zero-shot CoT, we describe that the recall, f1-score, MCC, and ROC_AUC of these two labeling techniques are not significantly different. However, due to the smaller P-value, it is clear that the precision of the Few-shot is significantly larger than that of the Zero-shot ZoT. Besides, the higher test-statistic values across all these 5 metrics indicate that the Few-shot has performed better than the Zero-shot CoT.

## Further discussion

In the subsequent section, we further analyze our primary results to extract more insightful observations.

### Performance of GPT labeling on best classifiers of human labels

**Fig 7:**
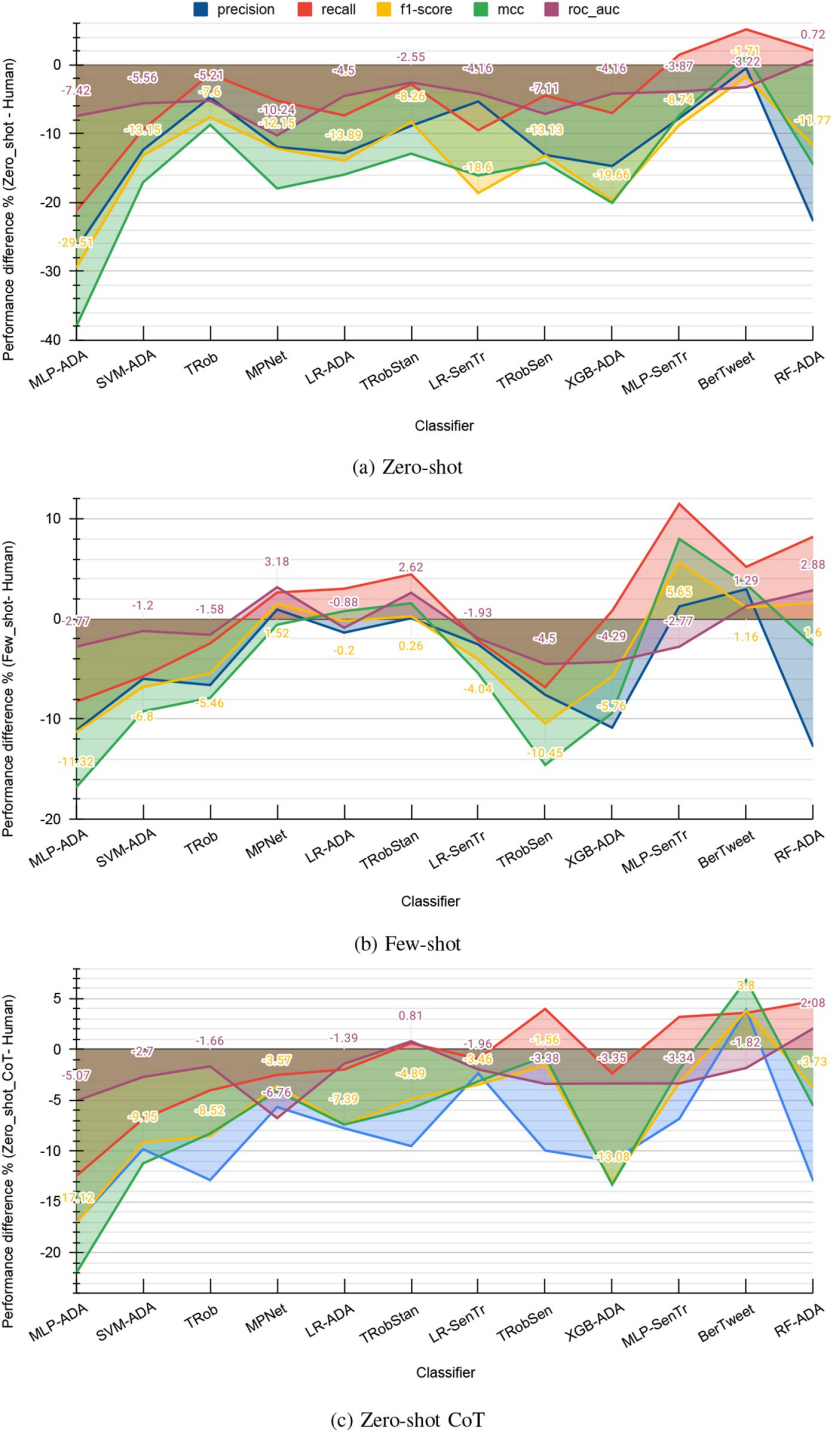
The percentage increase in performance compared to human-labeled data, observed across the top-performing classifiers of human labeling. (a) Zero-shot, (b) Few-shot, (c) Zero-shot CoT.

Referring to Table 2, it is evident that the baseline experiment showcased the highest performance from models, namely MLP-ADA, SVM-ADA, and TRob (Twitter Roberta) across a majority of metrics. In Fig 7, we visualize the percentage improvements in performance (improvement percentage = (GPT result—human result) * 100) achieved by GPT-based labeling techniques across the top 12 models that achieved the best f1-scores (f1 ≥ 0.70) with human labels. Additionally, on the graphs, we numerically labeled the differences in performance for f1-score and ROC_AUC, two crucial metrics for evaluating an imbalanced multi-class classification task [38, 39]. In these graphs, the positive regions signify enhanced performance, while the negative regions reflect performance that failed to achieve the standards set by human labeling.

When comparing with Few-shot and Zero-shot CoT, the majority of the area in the Zero-shot category lies in the negative region, with a more substantial negative difference, reaching as low as -40.00%. Notably, BerTweet and TRobStan stand out as the top-performing models in the Zero-shot category, closely aligning with human labels across all metrics. In contrast, the performance of Few-shot occupies a larger positive area for many ML models. TRobStan and BerTweet emerge as the leading models, surpassing human labels through all the metrics, while MPNet, LR-ADA, and MLP-SenTr are a few other models performing at par with human labels. Among these models, BerTweet is highlighted as the best model for Zero-shot CoT labels, with only a minor decrease in ROC_AUC compared to human labels. Additionally, LR-SenTr and MPNet are two of the models with considerable performance.

However, it is essential to note that none of the GPT-4 techniques were able to match or surpass the human benchmark set by the top-performing three models, MLP-ADA, SVM-ADA, and TRob. Apart from that, out of all the labeling techniques, it is noteworthy that the percentages in the gap of recall and ROC_AUC between GPT and human labels are relatively lower compared to the other metrics. Moreover, similar to the literature that suggests MLP as one of the robust traditional classifiers on imbalanced datasets [40], we found MLP with ADA or Sentence Transformers produced better results when fine-tuned on human labels.

### The best classifiers of GPT-based labels

Table 4 lists the best-performed classifiers trained on GPT-based training labels, ordered by f1-score. Noticeably, the LLMs, such as BerTweet, TRob, TRobSen, and TRobStan which were pre-trained on Twitter datasets were among the top ten of all the three prompting techniques. MPNet, SVM-ADA, and LR-ADA embedding are the other classifiers commonly performed when trained on any GPT-based labeling set. Additionally, no traditional classifiers with Glove embeddings are within the best performances and all six combinations of them are listed within the ten worst-performed classifiers of all three GPT-based labeling methods. Moreover, we noticed Albert as the model gained the least performance over all the five metrics in all the three labeling approaches.

**Table 4:** Top classifiers trained on different GPT-based labeling sets based on f1-score.

Rank	Zero-shot	Few-shot	Zero-shot CoT
1	TRob	MPNet	BerTweet
2	BerTweet	TRobStance	MPNet
3	TRobStance	LR-ADA	TRobSentiment
4	SVM-ADA	MLP-SenTrans	SVM-ADA
5	MPNet	MLP-ADA	TRobStance
6	LR-ADA	SVM-ADA	LR-SenTrans
7	MLP-SenTrans	TRob	TRob
8	TRobSentiment	BerTweet	SVM-SenTrans
9	GB-ADA	RF-ADA	MLP-ADA
10	SVM-SenTrans	LR-SenTrans	LR-ADA

### GPT performance above the benchmark

**Fig 8:**
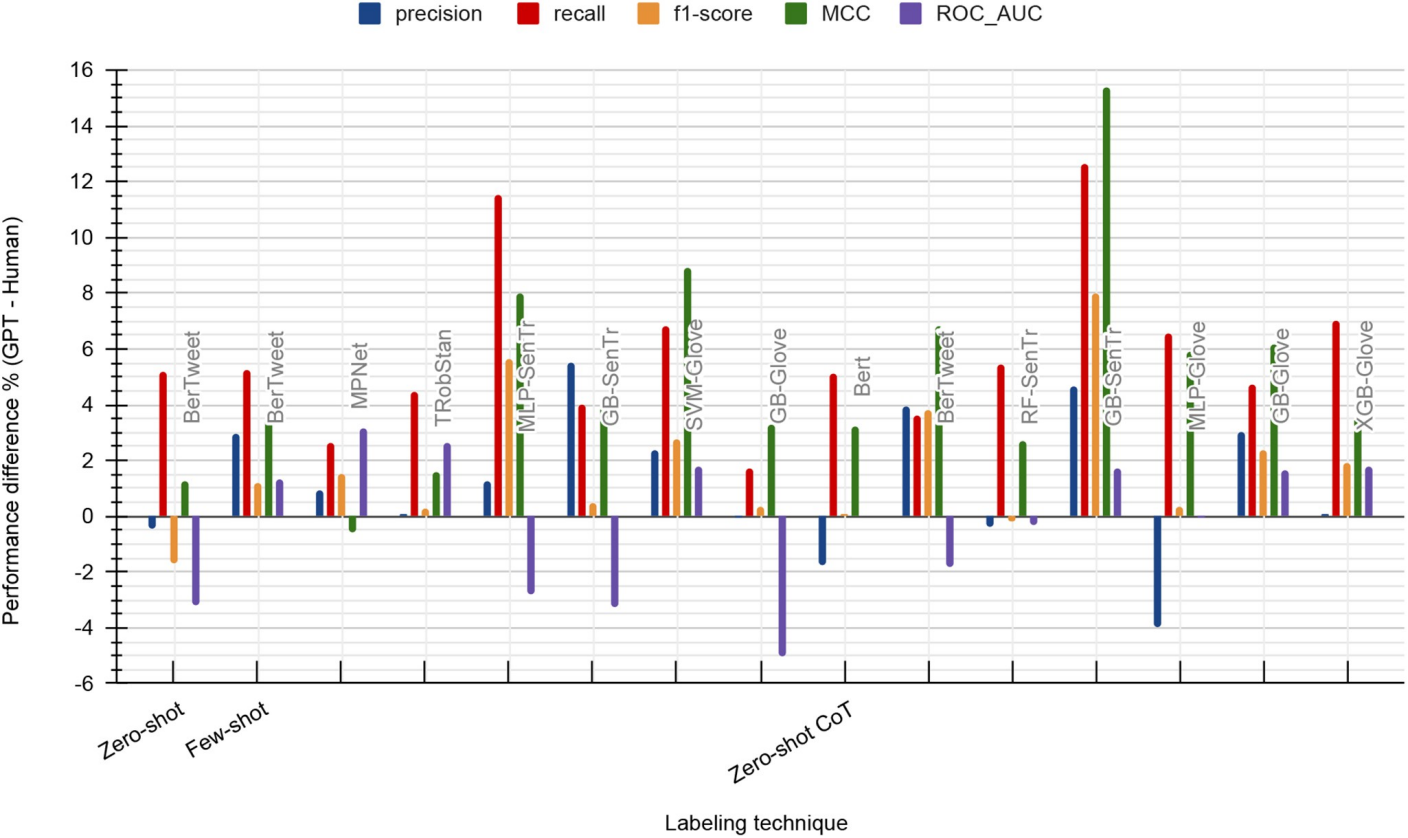
Performance analysis of classifiers trained on GPT-4’s labeled datasets, which outperformed ground truth labels.

In this section, we focus on highlighting the classifiers trained using GPT-4’s labeled datasets that have exceeded the performance of ground truth labels. Based on the cells highlighted in Table 2, we selected the models that excelled in at least four out of five metrics compared to the baseline. However, with Zero-shot labeling, we observed improved performance in a maximum of three out of five key metrics (Please note that Table 2 displays the values rounded up to two decimals. Hence, a highlighted cell with equal performance in Table 2 can be displayed as a negative difference percentage of less than 0.5 in Fig 8). The percentages of performance gaps between GPT-4 techniques and human labels of these models are presented in Fig 8.

In Zero-shot method, only BerTweet satisfies this criterion. On the other hand, Few-shot labeling has exhibited enhanced performance across seven models, with three of them being LLMs. Out of the seven classifiers that outperformed during Zero-shot CoT, the one using GB with Sentence Transformer embedding emerged as the best, surpassing human label performance. It is worth noting that there were no classifier-embedding combinations using ADA embedding, despite its presence among the top-performing classifiers based on human labels. Additionally, BerTweet consistently delivered impressive results across all three GPT-4 labeling techniques.

Finally, it is noteworthy to compare the models presented in this section and the best classifiers based on human labels in Fig 7 to understand how GPT-4 labeling techniques have achieved or exceeded the high standards set by humans. While Zero-shot labeling failed to meet this threshold, four models in the Few-shot category; BerTweet, MPNet, TRobStan, and MLP-SenTr along with BerTweet in Zero-shot CoT, surpassed the best ground truth performances across various metrics.

### Improvements with Zero-shot CoT mechanism

**Fig 9:**
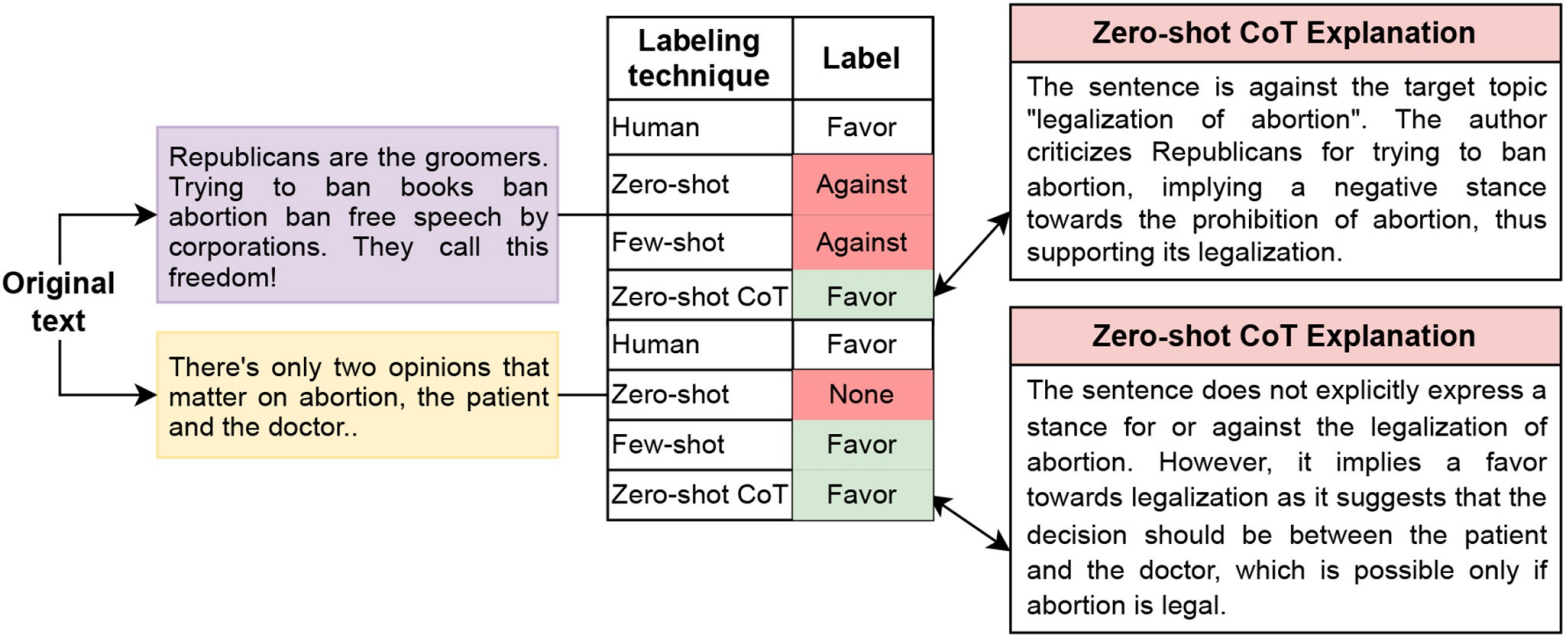
Two examples explaining the advantage of Zero-shot CoT over the basic Zero-shot prompting mechanism.

This approach has been implemented in generating answers to arithmetic, symbolic, and logical reasoning problems [23]. In this paper, we applied the Chain-of-Thoughts concept to comprehend and label social media texts, which exhibit their own unique characteristics. As mentioned, this prompting approach has the benefit of allowing the model to reassess its answer before determining the final label. Fig 9 shows a few examples of how GPT-4 has changed its final answer based on this re-thinking strategy.

In both examples, Zero-shot assigns an incorrect label. In contrast, in Zero-shot CoT, it reads its own explanation and corrects the label. Both explanations clarify how GPT-4 initially generates incorrect answers for Zero-shot prompts. For instance, in the second explanation, it first states that the sentence does not explicitly express a stance on the legalization of abortion, leading to a ‘none’ label. However, it later expands its explanation, understanding an alternative viewpoint, and correctly labels it as ‘favor’.

### Limitations and future work

It is worth acknowledging that there is room for improvement in the quality of data annotated by GPT-4 when compared to human-annotated data. This study has some limitations, including a smaller dataset size and the use of a single dataset for stance detection, which may not fully capture the complexities of labeling social media text in stance classification, requiring domain-specific expertise. Since the GPT-4 model used in this study was fine-tuned only on data up to 2021, it likely lacks knowledge or understanding of this specific incident related to abortion legalization that occurred in 2022. This could result in misinterpretations or inaccurate labeling of tweets, leading to biased or unreliable labels. Furthermore, GPT models are highly sensitive to prompts and continually evolving, hence reproducibility of results must be considered. Our future work will involve expanding to multiple datasets and investigating the impact of the number of examples in Few-shot learning. Additionally, a comprehensive examination of GPT model robustness will be valuable, given that our approach employed fixed prompts and was resource-intensive due to the repeated execution of prompts to balance robustness and creativity in label generation. Furthermore, in the future, we could consider retraining a GPT model that has been trained on more recent data to enable better understanding and contextualization of tweets specific to this and more recent events.

## Conclusion

Annotating social media text is a challenging task for humans due to the brevity, informality, and embedded socio-cultural opinions and perceptions in these texts where insufficient context understanding can result in low-quality annotations. To address this challenge, this study explores the potential of the GPT-4 model as an effective tool for labeling social media text, selecting stance labeling as the problem due to its relative complexity among other NLP tasks. We compare its performance across three prompting techniques, Zero-shot, Few-shot, and Zero-shot Chain-of-Thoughts (CoT) with human-labeled data. By observing the label distribution and the extent of alterations made to the original labels, it became evident that the Few-shot approach, followed by the Zero-shot CoT method, exhibits a higher degree of similarity to human experts in the assignment of labels to tweets. The overall results gained through 26 classifiers highlight the superiority of human labels, achieving higher performance across numerous metrics. However, several machine learning models fine-tuned on both Few-shot and Zero-shot CoT labels demonstrate enhanced or competitive individual performance, showcasing their ability to match human annotators in this task. Remarkably, we noticed that BerTweet has exhibited outstanding performance across all three labeling techniques. The Large Language Models, pre-trained on Twitter data, such as BerTweet, Twitter Roberta (TRob), Twitter Roberta Stance (TRobStan), and Twitter Roberta Sentiment (TRobSen), generally yield better results when fine-tuned on GPT-4-based labels or human labels. Furthermore, Zero-shot CoT demonstrated its strength compared to basic Zero-shot methods in labeling social media text for stance classification. Moreover, it competes effectively with the resource-intensive Few-shot approach, highlighting its capacity to produce reliable results without relying on labeled data samples. We anticipate that our findings will shed light on the utility of the GPT-4 model, for automating data annotation in social media text and inspire future research aimed at improving the quality and dependability of generated data.

## Supporting information

S1 Table Large language models, their pre-trained versions, and pre-trained datasets. (PDF)

S1 File The equations representing the evaluation metrics used in the study. (PDF)
