# Supplementary material for: GPT-4 as an X data annotator: Unraveling its performance on a stance classification task
Source: PLoS One. 2024 Aug 15;19(8):e0307741. doi: 10.1371/journal.pone.0307741 (PMC11326574; doi:10.1371/journal.pone.0307741)
Supplement: S1 Table — (PDF) [file pone.0307741.s001.pdf]

**Model**	**Version**	**Pre-trained dataset**
Bert	bert-base-uncased	BooksCorpus (800M words) and English Wikipedia (2,500M words)
Albert	albert-base-v2	Same dataset of Bert
Deberta	microsoft/deberta-base-mnli	English Wikipedia (12GB), BookCorpus (6GB), OpenWebText (public Reddit content of 38GB), and STORIES (a subset of CommonCrawl of 31GB).<br>The size of the total data set after deduplication is about 78G.
BerTweet	vinai/bertweet-base	850M English Tweets containing 845M Tweets streamed from 01/2012
MPNet	microsoft/mpnet-base	160GB data from Wikipedia, BooksCorpus, OpenWebText, CC-News and Stories.
Roberta	cardiffnlp/twitter-roberta-base-2022-154m	154M tweets of general conversations between 2018-01 and 2022-12.
Roberta	cardiffnlp/twitter-roberta-base-sentiment-latest	60M tweets were obtained by extracting a large corpus of English tweets (using the automatic labeling provided by Twitter).
Roberta	cardiffnlp/twitter-roberta-base-stance-abortion	
