# Supplementary material for: GPT-4 as an X data annotator: Unraveling its performance on a stance classification task
Source: PLoS One. 2024 Aug 15;19(8):e0307741. doi: 10.1371/journal.pone.0307741 (PMC11326574; doi:10.1371/journal.pone.0307741)
Supplement: S1 File — (PDF) [file pone.0307741.s002.pdf]

1 Precision and Recall

$$Precision_k = \frac{TP_k}{TP_k + FP_k} \quad (1)$$

$$Recall_k = \frac{TP_k}{TP_k + FN_k} \quad (2)$$

Where,

- True Positive (TP) is the correctly classified samples for the class k
- False Positive (FP) and False Negative (FN) are the incorrectly classified samples on the predicted and actual classifications of the class k

2 Macro Precision, Macro Recall and Macro F1-Score

$$MacroAveragePrecision(MP) = \frac{\sum_{k=1}^{N} Precision_k}{N} \quad (3)$$

$$MacroAverageRecall(MR) = \frac{\sum_{k=1}^{N} Recall_k}{N} \quad (4)$$

$$MacroF1 - Score = 2 * \frac{MP * MR}{MP^{-1} + MR^{-1}} \quad (5)$$

Where,

- N is the total number of classes in the dataset

3 Matthew's correlation coefficient (MCC)

Based on the number of classes N, and confusion matrix C with actual results on rows (i) and predicted results on columns(j).

$$MCC = \frac{c * s - \sum_{k}^{N} P_k * t_k}{\sqrt{(s^2 - \sum_{k}^{N} P_k^2)(s^2 - \sum_{k}^{N} t_k^2)}} \quad (6)$$

Where,

- $c = \sum_{k}^{N} C_{kk}$ the total number of elements correctly predicted
- $s = \sum_{i}^{N} \sum_{j}^{N} C_{ij}$ the total number of elements
- $P_k = \sum_{i}^{N} C_{ki}$ the number of times that class k was predicted (column total)
- $t_k = \sum_{i}^{N} C_{ik}$ the number of times that class k truly occurred (row total)
